# Failure of Israeli pediatric residency curricula to cover child development and special education issues: results of a national survey on levels of knowledge

**DOI:** 10.1186/s13584-021-00480-y

**Published:** 2021-09-21

**Authors:** Itay Tokatly Latzer, Zachi Grossman, Nimrod Sachs, Orr Yahal, Daniel Even-Zohar, Lior Carmon, Hadar Flor-Hirsch, Amit Ringel, Christopher Fady Farah, Moran Avni-Maskit, Yael Leitner

**Affiliations:** 1 Child Development Institute, The Dana-Dwek Children’s Hospital, Tel Aviv Medical Center, Tel Aviv, Israel; 2 Sackler Faculty of Medicine, Tel Aviv University, Tel Aviv, Israel; 3 Pediatric Clinic, Maccabi Healthcare Services, Tel Aviv, Israel; 4 Adelson School of Medicine, Ariel University, Ariel, Israel; 5 Department of Pediatrics C, Schneider Children’s Hospital, Rabin Medical Center, Petach Tikvah, Israel; 6 Department of Pediatrics A, Edmond and Lilly Safra Children’s Hospital, Chaim Sheba Medical Center, Tel-Hashomer, Israel; 7 Department of Pediatrics, Hadassah Medical Center, Jerusalem, Israel; 8 Department of Pediatrics, Faculty of Medicine, Soroka University Medical Center, Beer-Sheva, Israel; 9 Pediatric Neurology Unit, Assaf Harofeh Medical Center, Rishon Lezion, Israel; 10 Department of Pediatrics, Kaplan Medical Center, Rehovot, Israel; 11 Department of Pediatrics, Ruth Rappaport Children’s Hospital, Rambam Health Care Campus, Rappaport Faculty of Medicine, Technion, Haifa, Israel; 12 Department of Pediatrics, HaEmek Medical Center, Afula, Israel

**Keywords:** Child development, special education, pediatrics, training, residency

## Abstract

**Background:**

There is an increasing prevalence of developmental difficulties among Israeli children. We aimed to assess whether pediatricians are equipped to diagnose and manage them.

**Methods:**

We assessed the knowledge of basic child development issues and availability of services and content of special education systems among a randomly selected national sample of residents and senior Israeli pediatricians. This was done via an 70-itemed survey developed especially for this study which consisted of seven main subjects: developmental milestones, global developmental delay, autism spectrum disorder, attention deficit hyperactivity disorder, protocol for referring to a child development institute, availability and facilities of special education systems, and medical conditions associated with developmental delay.

**Results:**

A total of 310 pediatricians (an 86 % usable response rate) participated. The total median knowledge score was 32.1 % (IQR 17.8–53.5 %). Knowledge was significantly better among senior pediatricians (*p* < .001), those working in an office-based setting (*p* < .001), and those who were parents (*p* < .001) or had a family history of a developmental condition (*p* = .003). Most responders (94 %) felt that their resident training in child development was inadequate, and that they do not have sufficient access to resources and guidelines about child development and special education systems (80 %).

**Conclusions:**

The gap in knowledge on topics of child development and special education systems among Israeli pediatricians stems from inadequacies in the current curricula of pediatric residencies. The alarmingly low scores of our survey on these issues call for prompt revamping of the syllabus to include them.

**Supplementary Information:**

The online version contains supplementary material available at 10.1186/s13584-021-00480-y.

## Background

There are currently 3154 licensed pediatricians in Israel, 73 % (2306) of whom are younger than 65 years. The national ratio of active pediatricians per 1000 children is 0.768 [1, 2]. Twenty-three medical centers, six of which are tertiary, are accredited for operating a pediatric residency program in Israel, and there are currently 645 pediatric residents in training [3]. The official curricula of the 54-month long pediatric residency in Israel includes 33 months in a general pediatric department, six months in a neonatal intensive care unit, six months in a pediatric outpatient clinic, one month in a pediatric emergency room, two elective months in one of many pediatric specialties, and six months of basic research [4].

The official academic syllabus of the residency states that certified pediatricians should be knowledgeable of basic subject matters related to child development and the local special education systems available to children from birth to 17 years. Knowledge should encompass milestones of development, the major entities of child development [global developmental delay (GDD), autism spectrum disorder (ASD), intellectual disability (ID), language disorders and attention deficit hyperactivity disorder (ADHD)], the structure and services provided by Child Development Institutes, and the procedure of placement in a special education system and therapies/services, such as physical, speech, and occupational therapy [5]. Requirement of this knowledge notwithstanding, there is no designated minimal timeframe of training in child development during residency. Rather, this knowledge it is usually acquired en site from senior pediatricians in general pediatric departments, neonatal intensive care units, outpatient clinics, and or on-the-job learning in randomly assigned daily shifts that residents perform in community-based well-baby care clinics. The responsibilities of these latter clinics are the provision of preventive care to children from birth until five years of age, which includes vaccinations and periodically performed development screens done by general physicians/pediatric residents/senior pediatricians [6]. Pediatric residents attending these clinics do not undergo formal training in child development beforehand.

A total of 98,332 children were placed in special education systems in Israel in 2019, which corresponds to 5.5 % of all the children enrolled to the education systems overall [7]. The absolute majority children who are placed in special education systems do so after going through a diagnostic and assigning process completed by a child development specialist. Pediatricians are expected to serve as the initial advisors and counsellors for parental and educational staff concerns regarding child development issues. Thus, knowledge of these topics is crucial in their everyday practice both in the office-based and hospital settings. The current state of affairs is that Child Development Institutes in Israel are overwhelmed by unnecessary referrals from pediatricians. In our belief, this is a result of a lack of professional knowledge. The extent of pediatrician’s knowledge of current practices of child development and special educations systems has never been assessed. In this study, we aimed to evaluate self-reported knowledge of this field, and identify any major existing gaps that should have been covered in pediatric residency curricula.

## Methods

### Study design and settings

Primary care and hospital-based pediatricians and pediatric residents were recruited from November 2020 to January 2021 in 12 hospitals and through an organized and inclusive network of licensed primary-care pediatricians who practice in the four Israeli healthcare providers. The invitation to participate in the study was sent as a link by a representative physician in every hospital and healthcare system. A cover letter described the purpose of the survey and ensured anonymity of the responder. Additionally, participants were asked to be trustworthy by not seeking answers elsewhere but from their own knowledge, in order to maintain the integrity of the results. Proceeding from the cover letter to the survey contents implied consent to participate in the study. Compensation was not offered for survey completion. Responders who had not completed their residency training in Israel were excluded, as were whose forms had missing items. The study protocol was approved by the Institutional Review Board of the Tel Aviv Medical Center.

### Measures

We developed a 70-itemed and 15-25-minute web-mediated Qualtrics survey especially for this study. Pre-distribution of the survey, it was pilot-tested on an accessible sample of 10 pediatricians from diverse profiles and practice settings and revised according to their remarks and suggestions.

The first part of the survey collected demographic data that included sex, current age, place of birth, year of immigration, and information on medical education, current practice setting, years of practice as a pediatrician, type of subspecialty, number and age of own children, and whether there is a first-degree relative with developmental disorders. In the next section, the pediatricians were queried about their knowledge of general child development-related subjects (developmental milestones, GDD, ASD, ADHD, and medical conditions associated with developmental delay] and topics relating to the local special education system (the process of referral of a child to a child development institute and the services provided by the different local special education systems).

The respondents were then specifically asked if they believed that their training in child development was satisfactory, if they felt that they have sufficient access to child development resources and guidelines, and how often they encounter children who present with developmental issues. The correct answers to the subject matters on child development were based on the Nelson textbook of Pediatrics [8], which is the official textbook used for pediatric training by the Scientific Council of the Israeli Medical Association [5]. The answers to the topics of the special education systems were based on the relevant position paper and official clinical guidelines of the Israeli Medical Association [9]. Table 1 lists the selected questions used in the knowledge questionnaire. The full version of the questionnaire can be found as supplementary material.

**Table 1:** Examples of Selected Questions in the Knowledge Survey

• Developmental milestones	
o What is the average age for a child to crawl? o How many words should an 18-month-old be able to say? o What is the average age when a child should be able to exhibit ‘functional play’?	
• Global developmental delay (GDD)	
o What is the definition of GDD? o What comprises the basic evaluation of every child presenting with GDD? o How is the developmental quotient (DQ) calculated?	
• Autism spectrum disorder (ASD)	
o What are the two criteria that must exist to establish the diagnosis of ASD? o What determines the severity of ASD? o Is it possible to diagnose an individual with both ASD and intellectual disability (ID)?	
• Attention deficit hyperactive disorder (ADHD)	
o What are the three primary types of ADHD? o What is the initial recommended treatment for preschool ADHD? o What is the first-line pharmacologic treatment for individuals above the age of 6 years who have been diagnosed with ADHD?	
• Referral to a Child Development Institute	
o Which developmental screening tests are you familiar with? o Where should we refer a child with behavioral/disciplinary problems? o What are the age and weight cutoffs for referring preterm babies to a child development institute?	
• The special education systems o Which types of special education systems are you familiar with? o Which special education system would be suitable for a child with GDD? o When a “placement committee” decides to place a child in a special education system, must his legal guardians comply or can they choose to ignore the committee’s decision?	
• Medical Conditions Associated with Developmental Disorders o Name three syndromes caused by a chromosomal abnormality for which developmental disability is part of the presentation? o Which intrauterine infection can lead to a developmental disability? o What is the most common genetic syndrome leading to ID in males?	

ADHD- Attention deficit hyperactive disorder; ASD- Autism spectrum disorder; DQ- Developmental quotient; GDD- Global developmental delay; ID- intellectual disability

### Statistical analysis

Data were analyzed with SPSS Statistics (IBM SPSS Statistics, Version 25, 2017, IBM Corp, Armonk, NY, USA). A two-sided *p* value < 0.05 was considered statistically significant. Categorical variables were reported as frequency and percentages. The distribution of continuous variables was evaluated by a histogram. Since all continuous variables were non-normally distributed, they were reported as median and interquartile ranges (IQRs) in addition to means and standard deviations. Spearman’s correlation coefficient and the Mann-Whitney test were applied to study the association between continuous and categorical predictors and the studied scores. The total knowledge score was also non-normally distributed, and it was transformed into categorical variables using the upper quartile value as the threshold value. The Chi-square and Mann-Whitney tests were applied to study the association between the continuous and categorical predictors and the transformed total score. Multivariable analysis was performed by means of logistic regression. Variables that were found to be significantly associated with high total knowledge of child development and special education (*p* ≤ .05) were considered as candidates for multiple logistic regression. Adjusted odd ratios (OR) and 95 % confidence intervals (CI) were reported. Goodness-of-fit of the model was estimated by a Hosmer-Lemeshow test.

## Results

A total of 363 pediatricians received the survey, out of whom 310 competed it, yielding a response rate of 85 %. Fifty-three responders were excluded: 45 for incomplete surveys, seven for having completed their pediatric residency abroad, and one who had completed a child development fellowship. Out of the 310 respondents, 133 (43 %) were residents and 177 (57 %) were senior pediatricians [182 (59 %) females] completed the survey. Their demographic characteristics are presented in Table 2. The median completion time of the 310 valid surveys was 19.6 (IQR 8.1–25.4) minutes.

**Table 2:** Demographic characteristics of the pediatricians participating in the study

Characteristic	n = 310 (100 %)
Sex Male Female	128 (41 %) 182 (59 %)
Age, years (median, IQR)	38 (34–46)
Seniority Resident Consultant	133 (43 %) 177 (57 %)
Current work setting Hospital Primary care	196 (63 %) 114 (37 %)
Pediatric subspecialty Yes No	56 (18 %) 254 (82 %)
Being a parent Yes No	232 (75 %) 78 (25 %)
Developmental conditions in close family members Yes No	69 (22 %) 241 (78 %)

IQR- interquartile range

**Fig. 1:**
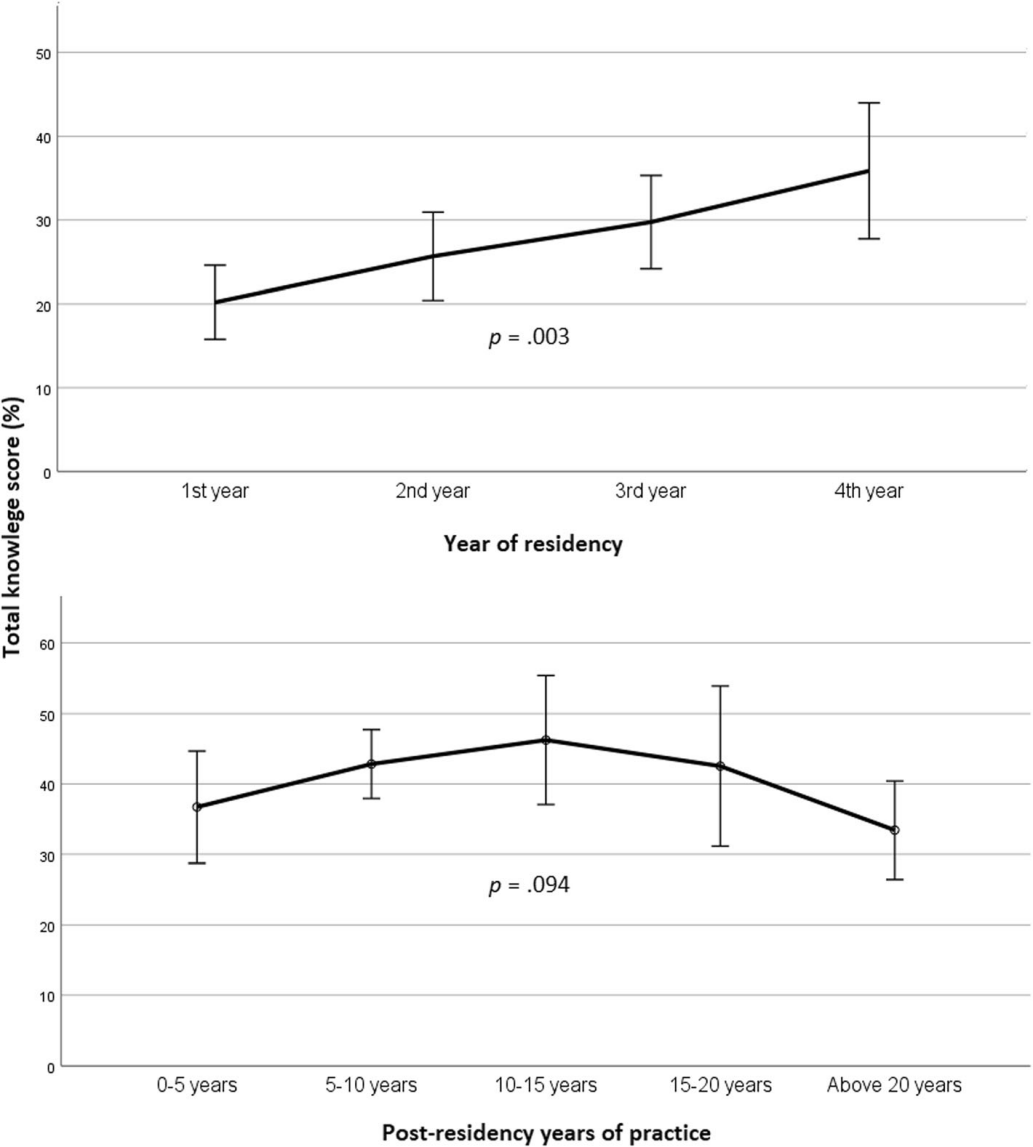
A line-plot displaying the significant advancement of knowledge along the years of residency (above) and the nearly consistent knowledge level between the different groups of post-residency years of practice (below)

The total median score of all the subject matters in the knowledge survey was 32.1 % (IQR 17.8–53.5 %) and the total mean score was 34.4 % ± 20.5 %. The highest mean and median scores were achieved in the item “medical conditions associated with developmental delay” [67.7 % ± 31.9 %, 66.6 % (IQR 33.3–100 %)] and the lowest in “special education systems” [13.6 % ± 19.4 %, 0.0 % (IQR 0.0–22.0 %)]. Knowledge scores for individual questions mostly ranged between 25 and 50 %, however, there were notable outlier questions. On the higher end, 89 % of respondents knew which is the most common intrauterine infection that can result in developmental delay and 73 % could name three conditions caused by chromosomal abnormalities that can present with developmental difficulties. There were more examples on the lower end: only 8 % knew at what age an infant is expected to exhibit functional play, only 9 % knew which special educations system is best suitable for a child with low-functioning ASD, only 10 % were able to give examples of developmental screening tests, and only 12 % knew at what age a toddler is expected to recognize and name at least five colors. There was a significant difference between the general knowledge of senior pediatricians and pediatric residents (*p* < .001). This also held true for each of the separate subject matters, with the exception of knowledge about medical conditions associated with developmental delay (*p =* .972). With the advancement in residency years, knowledge improved (*p* = .003) (Fig. 1). There was no correlation between age and an increment of knowledge level among senior pediatricians (*p =* .470). This also held true when differences between the groups of 0–5, 5–10, 10–15, 15–20 and > 20 post-residency years of practice were assessed (*p* = .094) (Fig. 1). Individuals with at least one child of their own had a significantly higher general knowledge than those without children (*p* < .001). There were no meaningful differences between the knowledge of female and male pediatricians with children (*p* = .752). Having a close family member with developmental conditions was also significantly associated with a higher level of knowledge (*p* = .001). Pediatricians working in an office-based setting were significantly more knowledgeable than those working primarily in hospitals (*p* < .001), and pediatricians with a subspecialty showed a significantly higher level of general knowledge than those without one (*p =* .007). There were no significant sex-related differences in overall knowledge (*p =* .112), in whether or not physicians were graduates of foreign medical schools (and performed their pediatric residency in Israel) (*p =* .087), or in whether residency was or was not performed in a tertiary medical center (*p =* .122). Table 3 summarizes the main responses of the different groups of pediatricians relating to knowledge of child development and of special education systems.

**Table 3:** Pediatricians’ knowledge scores* of the specific subject matters of child development and the special education systems

	Total score	Developmental milestones	Global developmental delay	Autism spectrum disorder	Attention deficit hyperactivity disorder	Referral to special education	Special education systems	Medical conditions associated with dev. delay
Total mean score Total median score (IQR)	34.4 ± 20.5 32.1 (17.8–53.5)	36.9 ± 24.3 40.0 (16.0–60.0)	24.0 ± 27.8 20.0 (0.0–40.0)	31.4 ± 29.6 25.0 (0.0–50.0)	47.0 ± 41.7 50.0 (0.0–80.0)	39.6 ± 30.2 40.0 (20.0–60.0)	13.6 ± 19.4 0.0 (0.0-22.2)	67.7 ± 31.9 66.6 (33.3–100)
Sex Male Female *p*-values	32.2 ± 19.7 36.0 ± 21.0 0.112	32.5 ± 22.7 40.1 ± 24.9 **0.007**	23.2 ± 27.4 24.6 ± 28.2 0.679	33.7 ± 30.1 29.8 ± 29.1 0.244	50.1 ± 44.3 44.9 ± 39.7 0.280	37.0 ± 29.5 41.4 ± 30.6 0.208	11.2 ± 17.4 15.3 ± 20.6 0.072	67.9 ± 31.1 67.5 ± 32.5 0.917
Seniority Resident Consultant *p*-values	27.3 ± 16.6 39.7 ± 21.6 **< 0.001**	27.9 ± 21.7 43.7 ± 23.9 **< 0.001**	16.3 ± 23.8 29.8 ± 29.3 **< 0.001**	28.2 ± 28.4 35.7 ± 30.6 **0.028**	41.6 ± 33.8 51.1 ± 46.4 **0.046**	28.7 ± 26.3 47.7 ± 30.5 **< 0.001**	6.2 ± 12.6 19.2 ± 21.7 **< 0.001**	67.6 ± 26.5 67.7 ± 35.5 0.972
Current work setting Hospital Primary care *p*-values	30.5 ± 17.6 41.2 ± 23.3 **< 0.001**	33.0 ± 22.6 43.7 ± 25.6 **< 0.001**	18.7 ± 24.8 33.1 ± 30.4 **< 0.001**	27.1 ± 27.3 33.9 ± 30.6 **0.053**	41.7 ± 35.8 56.3 ± 49.1 **0.003**	32.7 ± 28.3 51.4 ± 29.9 **< 0.001**	8.6 ± 15.4 22.2 ± 22.5 **< 0.001**	67.3 ± 30.1 68.4 ± 34.8 0.776
Pediatric subspecialty Yes No *p*-values	41.1 ± 21.2 33.0 ± 20.1 **0.007**	47.2 ± 24.7 34.7 ± 23.6 **< 0.001**	28.9 ± 28.5 22.9 ± 27.6 0.149	30.8 ± 31.6 31.5 ± 29.2 0.857	49.6 ± 42.4 46.5 ± 41.6 0.615	44.6 ± 30.2 38.5 ± 30.2 0.170	18.6 ± 22.6 12.5 ± 18.5 **0.034**	70.8 ± 35.9 67.0 ± 31.0 0.424
Being a parent Yes No *p*-values	38.3 ± 21.4 23.0 ± 11.6 **< 0.001**	42.6 ± 24.3 20.2 ± 14.9 **< 0.001**	29.5 ± 29.2 7.6 ± 13.7 **< 0.001**	28.9 ± 29.1 38.7 ± 29.7 **0.011**	48.3 ± 44.9 43.3 ± 30.1 0.358	44.4 ± 30.8 25.1 ± 23.1 **< 0.001**	16.7 ± 20.6 4.4 ± 10.9 **< 0.001**	67.0 ± 34.3 69.6 ± 23.5 0.541
Developmental conditions in family Yes No *p*-values	41.5 ± 24.1 32.4 ± 18.9 **0.001**	45.5 ± 25.9 34.5 ± 23.3 **0.001**	32.1 ± 31.2 21.7 ± 26.4 **0.006**	32.6 ± 31.8 31.1 ± 28.9 0.713	52.4 ± 45.1 45.5 ± 40.6 0.226	48.4 ± 32.2 37.0 ± 29.2 **0.006**	20.6 ± 23.0 11.6 ± 17.8 **0.001**	68.1 ± 36.7 67.6 ± 30.4 0.912

*Scores are presented means and standard deviations of percentages unless mentioned otherwise. **Bold** indicates significant

**Fig. 2:**
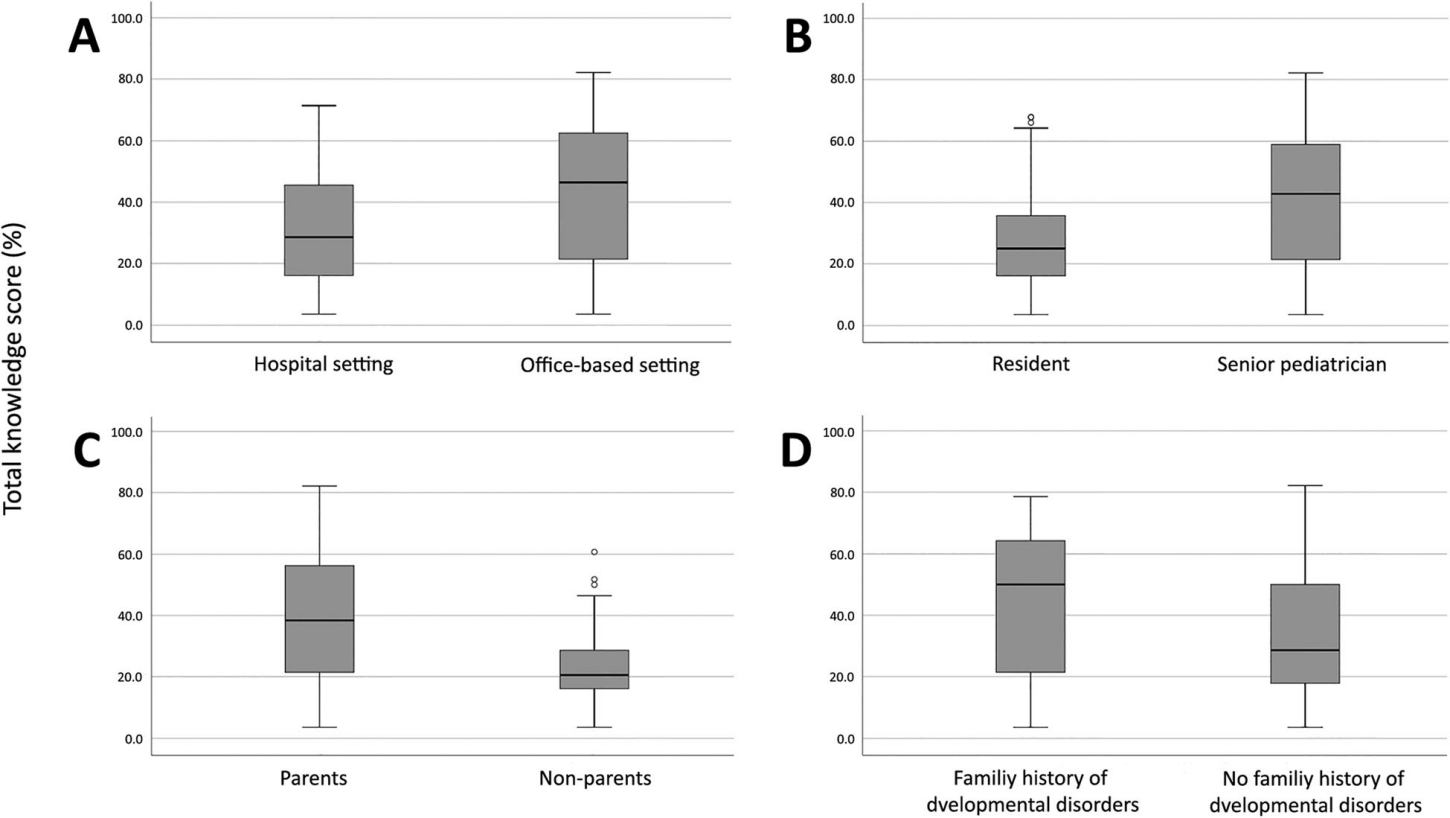
A box-plot depiction showing significantly higher total knowledge score percentages in (**A**) pediatricians working primarily in an office-based setting versus those in a hospital setting, (**B**) senior pediatricians versus pediatric residents, (**C**) pediatricians who are parents versus those who are not, and (**D**) pediatricians with a family history of developmental conditions

A comparison between the upper quartile and the lower three quartiles of total knowledge revealed consistency of almost all of the queried areas. The upper quartile of knowledge included significantly more senior pediatricians (*p* < .001), primary care pediatricians (*p* < .001), pediatricians who were parents (*p* < .001), and pediatricians who had a family history of developmental disorders (*p* < .001). Unlike the findings of the initial analyses, pediatricians with a subspecialty were not significantly more knowledgeable (*p* = .143). Table 4 presents the upper- and lower-third quartiles of total knowledge score percentages of child development and the special education systems among pediatricians. Figure 2 displays box plots of the significant total knowledge score percentages.

**Table 4:** Upper- and lower-third quartiles of knowledge scores of child development and the special education systems among pediatricians

	Upper quartile N = 71 (23 %)	Lower three quartiles N = 239 (77 %)	*p* value
Sex Male Female	24 (34 %) 47 (66 %)	104 (43 %) 135 (57 %)	0.170
Seniority Resident Consultant	12 (17 %) 59 (83 %)	121 (51 %) 118 (49 %)	**< 0.001**
Current work setting Hospital Primary care	24 (34 %) 47 (66 %)	172 (72 %) 67 (28 %)	**< 0.001**
Pediatric subspecialty Yes No	17 (24 %) 54 (76 %)	39 (16 %) 200 (84 %)	0.143
Being a parent Yes No	70 (99 %) 1 (1 %)	162 (68 %) 77 (32 %)	**< 0.001**
Developmental conditions in family Yes No	31 (44 %) 40 (56 %)	38 (16 %) 201 (77 %)	**< 0.001**

**Bold** indicates significant

The survey participants reported that they encounter children with developmental difficulties almost on every workday (n = 27, 8.7 %), once a week (n = 137, 44.1 %), twice a week (n = 69, 22.2 %) and almost never (n = 78, 25.1 %). The vast majority (n = 294, 94.5 %) of the respondents stated that their training in child development and special education was insufficient. Furthermore, the majority (n = 250, 80.4 %) reported not having easy access to clear-cut guidelines and to community resources in the field of child development and special education.

## Discussion

We conducted a national survey of Israeli pediatricians in order to quantify the general level of familiarity with several aspects of child development and the special education systems. The overall median score of the knowledge survey of the 310 respondents was 32.1 % which testifies to crucial gaps of knowledge.

Pediatric training programs are responsible of providing residents the educational clinical circumstances they need in order to prepare them for their future responsibilities, but the knowledge and skills attained during residency are mainly in the context of inpatient care. After completing residency, most pediatricians pursue a career in an office-based primary care setting in which developmental and special education issues arise more frequently than within hospital walls. This would explain why pediatricians working solely in a primary care setting exhibited a higher level of knowledge, apparently acquired by necessity after residency. There is no argument that it is important for young physicians to be exposed to acute and/or unique medical conditions during their residency. However, their training should also include exposure to conditions which are commonplace in office-based settings. We argue that proficiency of basic developmental assessments and knowledge of the basic structure of the special education systems should be ‘bread and butter’ as are other common pediatric conditions. More than one-half of the pediatricians in our study group reported that they encounter a child with a developmental issue once a week or more, with a vast majority of them feeling that their training in child development was lacking and that resources and guidelines in the field of child development were not easily accessible [10, 11]. This further highlights the magnitude of this problem, and that adequate knowledge of these conditions is integral to ensuring the accuracy of referrals to child development institutes and for the continuous management of these children. A related study assessing the knowledge of Israeli pediatricians in child development topics that was published in 2010, reported higher general scores (~ 70 %) compared to our results. However, this study had fewer respondents and included fewer questions [15]. Additionally, the questions were multiple choice which are arguably easier to answer. Despite this, and in support of our findings, the majority (64 %) of pediatricians who responded in that study also stated that they felt a need to strengthen their knowledge base on child development topics [12]. Importantly, relevant information pertaining to child development could be found in the formal health ministry website [13], implying that part of the problem could also result from a lack of motivation to search for it.

As would be expected, the levels of knowledge were significantly higher among senior pediatricians compared to residents, but when analyzing only the senior pediatricians’ population, it was not significantly increased with increasing age, nor between groups of post-residency practice years. This implies that there is little knowledge is acquired once a pediatrician completes residency training (Fig. 1). If post-residency education is not formalized and relies on self-directed education, it may lead to a plateau or a decrease in the level of knowledge. A study on the same topics as ours showed no difference between self-assessed knowledge of common neurological problems of pediatricians who had practiced more and less than 10 years [14].

Continuing medical education (CME) in the US helps physicians revive their knowledge base and stay updated. Comparable attempts are being made to face these challenges in Israel. The recently launched “Goshen Project” [15] is directed at strengthening the training of residents and senior pediatricians in developmental, behavioral, and psychosocial areas [11], but it is neither mandatory nor widely implemented so that few pediatric residents actually benefit from it. Since the pediatric residency in Israel is longer than in the US (4.5 years compared to 3 years), extending it in order to increase the exposure to community pediatrics is also not a practical option.

A number of studies have examined these issues in the US and Canada, but their healthcare systems are different from the Israeli mandatory universal healthcare system and their surveys had dissimilar contents [16–20]. Their overall conclusion, however, was consistent with ours: there is a pressing need to improve the education of pediatric residents in the field of child development. For example, the need for more training time in developmental pediatrics in the US has led the Residency Review Committee (RRC) of the Accreditation Council for Graduate Medical Education (ACGME) to mandate a 1-month block rotation in developmental behavioral pediatrics (DBP) since 1997 [21]. Curriculum guidelines for residency training in DBP have also been proposed [22]. Of note, is one study that examined the effects of such a change by way of a nationwide survey of pediatricians who completed their training before and after its implementation. It concluded that the overall comfort level of pediatricians in topics of DBP was unchanged [23]. This may imply that one month is not enough, or that the program was unsuitable.

The respondents in our study who were parents themselves and those who had a personal family history of a developmental condition had significantly higher levels of knowledge of those subjects. It is only natural to compare one’s own experiences when facing medical situations. Yet, the responsibility of being familiar and proficient in this highly prevalent field of pediatrics should not rely upon and be influenced by an individual’s own experiences, or biased thereby [24].

Incorporating a mandatory timeframe spent in a child development institute into the residency program curriculum will allow residents to be exposed not only to the work of the developmental pediatricians but also to the multidisciplinary work done with children by psychologists, social workers, physiotherapists, speech therapists, and occupational therapists. Even if the resident is unlikely to attain expertise in these fields, merely being familiarized with them is satisfactory. For example, It would allow for a knowledge of the indications for referral of a patient to the various developmental services, the tools used for diagnosing developmental conditions such as cognitive/psychological evaluations, the processes of referral to special education system and the social rights of a child & family with a developmental condition. These are no less important than knowledge of the major disorders of child development, such as ASD, GDD, and ADHD. It should be mentioned that the insights of our study had been discussed by medical opinion leaders in Israel over the past few years. Several reports observed that the prevalence of ASD and ADHD have risen dramatically in recent decades, but the exposure and education of these relevant topics in the training of pediatricians has not kept pace [25–27]. Noteworthy, as part of the efforts to address this issue, in 2013 the Israeli Ministry of Health established unique programs that train physicians to diagnose and treat ADHD.

Historically, the content of the pediatric residency program is Israel was entirely determined by hospital-based pediatricians. The lack of a sufficient workforce in most hospitals also made it necessary for residents to work primarily in the hospital wards as opposed to being exposed to other fields, such as developmental medicine. Recently, however, there has been a growing understanding that primary care is as important as secondary and tertiary care. The voice of community-based pediatricians is also being heard more prominently in forums of academia and in bodies which decide the curriculum of pediatric training. Taking this into consideration, and together with our findings that demonstrate a clear need to strengthen the competence of pediatricians in areas of child development, we hope some alarms will be set off and that change will ensue.

Interpretation of our findings should take into consideration several limitations. Since we inquired about the knowledge of pediatricians that underwent a residency program lacking formal training in child development, they cannot be generalized. Moreover, we cannot entirely rule that the insufficient knowledge in child development is not present in our respondents across other knowledge domains of general pediatrics, as this was not examined. Additionally, the study design relied upon the veracity of the respondents, and it is possible that pediatricians that felt a stronger relation to these topics were more likely to respond, resulting in a selection bias. However, this allowed us to achieve a relatively large sample size and reach individuals who might otherwise refuse to participate for fear their lack of knowledge would be exposed [28].

## Conclusions

Our study demonstrated that there is a significant knowledge gap in subject-maters of child development among Israeli pediatricians. Areas of child development are underrepresented in pediatric training, although they very often account for pediatric office visits. The implications of this study are that training of pediatric residents in child development should not be limited to on-the-job learning or to a few days/hours set aside by individual medical centers. Rather, policymakers are advised to re-evaluate and reconstruct the curriculum of pediatric residency in Israel, formalizing a unified designated period of time of child development training as an integral part of the pediatric residency program.

## Supplementary information

**Additional file 1**

## Data Availability

The datasets used and/or analyzed during the current study are available from the corresponding author on reasonable request.
