# Supplementary material for: Failure of Israeli pediatric residency curricula to cover child development and special education issues: results of a national survey on levels of knowledge
Source: Isr J Health Policy Res. 2021 Sep 21;10:45. doi: 10.1186/s13584-021-00480-y (PMC8454173; doi:10.1186/s13584-021-00480-y)
Supplement: Supplementary file 1 — Additional file 1 [file 13584_2021_480_MOESM1_ESM.docx]

**Child Development and Special Education Survey for Pediatricians**

**Demographics**

1. What is your sex?

2. How old are you?

3. Where did you study medicine?

4. Are you in residency or post residency?

5. Did you complete your pediatric residency locally or abroad?

6. In which hospital are you currently doing/did you complete your residency?

7. Where are you currently working?

8. How many years have you been a pediatrician (post-residency only)?

9. Do you have a sub-specialty?

10. What is your marital status?

11. Do you have children?

12. What are the ages of your children?

13. Do you have a first degree relative with a developmental disorder?

**Developmental milestones**

14. What are the main areas of child development?

15. What is the average age for a child to crawl?

16. What is the average age for a child to sit?

17. What is the average age for a child to be able to stand independently?

18. What is the average age for a child to walk independently?

19. What is the average age for a child to be able to stand on one leg?

20. What is the average age for a child to be able to catch a ball?

21. What is the average age for a child to develop a pincer grasp?

22. What is the average age for a child to reach out to grab an object?

23. At what age is the dominant hand usually determined?

24. What is the average age for a child to be able to draw a straight line?

25. What is the average age for a child to be able to build a tower of 2-3 cubes?

26. At what age does a social smile usually appear?

27. What is the average age for a child to be able to respond to their name?

28. What is the average age for a child to be able to respond to simple commands?

29. How many words should an 18-month-old be able to say?

30. What is the average age for a child to be able to construct a sentence with at least three words?

31. What is the average age when a child should be able to identify to 5 colors?

32. What is the average age when a child should be able to count to 10?

33. What is the average age when a child should be able to get dressed independently?

34. What is “object permanence”?

35. What is “functional play”?

36. What is the average age when a child should be able to exhibit ‘functional play’?

37. What is “parallel play”?

**Global Developmental Delay (GDD)**

38. What is the definition of GDD?

39. What is the definition of intellectual disability (ID)?

40. What comprises the basic evaluation for every child presenting with GDD?

41. How is the Developmental Quotient (DQ) calculated?

42. Which DQ value would lead to a diagnosis of ID?

**Autism Spectrum Disorder (ASD)**

43. What are the two criteria that must exist to establish the diagnosis of ASD?

44. What determines the severity of ASD?

45. What makes up the basic evaluation for every child presenting with signs of ASD?

46. Is it possible to diagnose an individual with both ASD and ID?

**Attention Deficit Hyperactive Disorder (ADHD)**

47. What are the three primary types of ADHD?

48. How is a diagnosis of ADHD made?

49. What is the initial recommended treatment for preschool ADHD?

50. What is the first-line pharmacologic treatment for individuals above the age of 6 who have been diagnosed with ADHD?

**Referral to a Child Development Institute**

51. What are the age and weight cut-offs for referring preterm babies to a child development institute?

52. Where should we refer a child to who presents with behavioral/disciplinary problems?

53. Where should we refer a child to who presents with expressive language difficulties?

54. Where should we refer a child to who presents with difficulties holding a pencil and cutting with scissors?

55. Which developmental screening tests are you familiar with?

**The Special Education System**

56. What comprises the routine process a child goes through prior to being placed in a special education system?

57. Which types of special education systems are you familiar with?

58. Which special education system would be suitable for a child with GDD?

59. Which special education system would be suitable for a child with low-functioning ASD?

60. Which special education system would be suitable for a child with severe ID caused by a genetic syndrome?

61. When a “placement committee” decides to place a child in a special education system, must his legal guardians comply or can they choose to ignore the committee’s decision?

62. Can children with genetic syndromes automatically be placed in a special education system?

63. Are there normal educations systems that can include children with developmental disabilities?

64. Which school-age special education systems do you know?

**Medical Conditions Associated with Developmental Disorders**

65. Name three syndromes caused by a chromosomal abnormality for which developmental disability is part of the presentation?

66. Which intrauterine infection can lead to a developmental disability?

67. What is the most common genetic syndrome leading to ID in males?

**General**

68. In your opinion, was your training in child development related issues satisfactory?

69. Do you feel you have a good access to child development services and guidelines?

70. How often (frequency/week) do you encounter a child with a developmental condition?
